# Outcome after Intracerebral Haemorrhage and Decompressive Craniectomy in Older Adults

**DOI:** 10.3390/neurolint16030044

**Published:** 2024-05-20

**Authors:** Thomas Kapapa, Stefanie Jesuthasan, Frederike Schiller, Franziska Schiller, Marcel Oehmichen, Dieter Woischneck, Benjamin Mayer, Andrej Pala

**Affiliations:** 1 Department of Neurosurgery, University Hospital Ulm, Albert-Einstein-Allee 23, 89081 Ulm, Germanyandrej.pala@uni-ulm.de (A.P.); 2 Department of Neurosurgery, Military Hospital Ulm, Oberer Eselsberg 40, 89081 Ulm, Germany; oehmichen45@hotmail.com; 3 Department of Neurosurgery, Hospital Landshut, Robert-Koch-Strasse 1, 84034 Landshut, Germany; 4 Institute for Epidemiology and Medical Biometry, University of Ulm, Schwabstrasse 13, 89075 Ulm, Germany

**Keywords:** demographic change, elderly, intracranial hypertension, brain edema, survival, mortality, morbidity, minimal invasive

## Abstract

Objective: There is a relationship between the incidence of spontaneous intracerebral haemorrhage (ICH) and age. The incidence increases with age. This study aims to facilitate the decision-making process in the treatment of ICH. It therefore investigated the outcome after ICH and decompressive craniectomy (DC) in older adults (>65 years of age). Methods: Retrospective, multicentre, descriptive observational study including only consecutive patients who received DC as the consequence of ICH. Additive evacuation of ICH was performed after the individual decision of the neurosurgeon. Besides demographic data, clinical outcomes both at discharge and 12 months after surgery were evaluated according to the Glasgow Outcome Scale (GOS). Patients were divided into age groups of ≤65 and >65 years and cohorts with favourable outcome (GOS IV–V) and unfavourable outcome (GOS I to III). Results: 56 patients were treated. Mean age was 53.3 (SD: 16.13) years. There were 41 (73.2%) patients aged ≤65 years and 15 (26.8%) patients aged >65 years. During hospital stay, 10 (24.4%) patients in the group of younger (≤65 years) and 5 (33.3%) in the group of older patients (>65 years) died. Mean time between ictus and surgery was 44.4 (SD: 70.79) hours for younger and 27.9 (SD: 41.71) hours for older patients. A disturbance of the pupillary function on admission occurred in 21 (51.2%) younger and 2 (13.3%) older patients (*p* = 0.014). Mean arterial pressure was 99.9 (SD: 17.00) mmHg for younger and 112.9 (21.80) mmHg in older patients. After 12 months, there was no significant difference in outcome between younger patients (≤65 years) and older patients (>65 years) after ICH and DC (*p* = 0.243). Nevertheless, in the group of younger patients (≤65 years), 9% had a very good and 15% had a good outcome. There was no good recovery in the group of older patients (>65 years). Conclusion: Patients >65 years of age treated with microsurgical haematoma evacuation and DC after ICH are likely to have a poor outcome. Furthermore, in the long term, only a few older adults have a good functional outcome with independence in daily life activities.

## 1. Introduction

The global burden of stroke is high and is represented by more than 80 million stroke survivors in 2016 [1]. The literature is ambiguous about changes in incidence and suggests a falling, steady, or rising incidence depending on the world region of interest [2,3,4]. Recent data show that, from a global perspective, the incidence and mortality rate rose alarmingly by around 5% between 2010 and 2017 [5]. The exact background to this increase is still unclear [6]. Nevertheless, the incidence of stroke shows a fixed relationship to age. The proportion of people <45 years of age with a stroke is between 5–10% in western countries and 19–30% in developing countries [1,7]. This means that with increasing life expectancy, the social and socio-economic challenges caused by stroke can grow globally [3].

Spontaneous, primary, non-traumatic intracerebral haemorrhage (ICH) is the second most common cause of stroke after ischemic stroke. It represents around 10–20% of all strokes [8]. However, there is also a relationship between the incidence of ICH and age. The incidence increases with age [9,10,11]. In an aging society due to demographic change, there is evidence that the incidence of ICH increases as the proportion of older people increases [12]. Due to the fact that societies are aging in many high-income countries and this in turn is related to social and socio-economic challenges, as well as challenges for in-hospital treatment and allocation of resources, this study aims to facilitate the decision-making process in the treatment of ICH [13,14,15]. This investigation focuses on the outcomes after ICH and decompressive craniectomy (DC) in older adults (>65 years of age). It is intended to enlarge the database for current and future evidence-based decisions.

## 2. Materials and Method

### 2.1. Study Design

This is a retrospective, multicentre, descriptive observational study, enrolling only patients who underwent DC between January 2005 and December 2021. The study was approved by the local ethics committee (No. 439/17). All included patients were initially treated at the three study centres or sent as a secondary transport from a different hospital.

### 2.2. Patients and Treatment Procedures

Level of consciousness before surgery was rated on the Glasgow Coma Scale (GCS) [16]. All consecutive patients who received DC as the consequence of intracerebral haemorrhage were included in the study during the described period. Standardised fronto-parieto-temporal craniectomy with temporo-basal osteoclastic enlargement and opening of dura mater was performed as described earlier [17]. The indication for DC was based on the clinical and radiological signs related to increased intracranial pressure due to ICH [18,19,20,21]. Specifically, the indication was determined by analogy with the treatment of primary or secondary DC in TBI. Primary DC was performed in patients with imaging evidence of intracranial space-occupying ICH (e.g., midline shift >5mm, compressed basal cisterns) and clinically comatose patients (GCS < 9 for <60 min) at the start of inpatient treatment. Secondary DC was performed to treat clinical deterioration in a previously awake patient (GCS < 9) and/or to treat increased intracranial pressure (>25 mmHg in >15 min) and failure of conservative management [21,22,23]. Large or mid-large, fixed pupils bilaterally as well as patients >80 years of age in coma on admission were considered disqualifiers for this procedure. The additive evacuation of ICH was performed after the individual decision of the neurosurgeon (lobar location of ICH, dimension of ICH, clinical status, etc.).

### 2.3. Data Collection and Outcome

Explorative assessment of clinical and radiological data of patients with ICH and DC was performed. Radiological characteristics of ICH such as the volume in cm^3^, the laterality, as well as the involvement of the dominant hemisphere and the resulting midline shift were recorded. The volume was calculated based on the initial imaging using the “Elements: Contouring 4.0” software (BrainLab, Munich, Germany). The location of the haemorrhage was classified according to the supra-tentorial lobes of the brain. In addition, a distinction was made between deep (basal ganglia and internal capsula) and lobar (cortical-subcortical areas) ICH [24]. Besides demographic data, clinical outcome at discharge and 12 months after surgery was evaluated according to the Glasgow Outcome Scale (GOS) [16]. The outcome data were provided either based on follow-up medical records or telephone interviews. Patients were divided into a cohort with favourable outcome, graded as GOS IV–V, and unfavourable outcome, defined as GOS Grade I–III. One patient was lost to follow-up because she did not reside in the country of the study.

### 2.4. Statistical Analysis

Explorative assessment by standard statistical methods was performed. The mean, standard deviation (SD), median, and range were reported in the case of quantitative parameters, absolute and relative frequencies for the qualitative parameters. The authors present results of the ordinal scaled GCS. These results are given in mean (SD) and median (range). The authors are aware that the magnitude of difference between each assigned number is not the same and therefore the mean (SD) is not the statistically appropriate expression of the centre of the values. Nevertheless, in many publications, the clinical centre point ranking of the GCS value is given with the mean, the median or, in more recent publications, with both for better comparison of the studies. We would like to follow this trend in this publication. Significance was set as *p* ≤ 0.05. Regression analysis was performed to identify the impact of the variables: age, time between symptom onset and surgery, as well as initial GCS on unfavourable outcome. All statistical tests were analysed using the IBM SPSS Statistics Software, Version 25 (IBM Corp., Armonk, NY, USA).

## 3. Results

In the specified period, 56 patients were treated for ICH with DC. The mean age was 53.3 (SD: 16.13) years, median 56 (7–89) years. The mean age of women was 54.6 (SD: 16.50) years with a median of 59 (7–71) years and that of men was 52.4 (SD: 16.05) years with a median of 50 (23–89) years (*p* = 0.247, Mann–Whitney U Test). The cohort was divided into 41 (73.2%) patients aged ≤65 years and 15 (26.8%) patients aged >65 years. (Table 1).

Fifteen (26.8%) patients died during the hospital stay. Ten (24.4%) in the group of younger patients (≤65 years) and 5 (33.3%) in the group of older patients (>65 years) died (*p* = 0.514, Fisher Exact Test). The mean time between the onset of symptoms and surgery was 39.6 (SD: 63.66) hours with a median of 13 (2–288) hours for the entire cohort. For younger patients (≤65 years), mean time was 44.4 (SD: 70.79) hours with a median of 14 (3–288) hours, and for older patients (>65 years), the mean time was 27.9 (SD: 41.70) hours with a median of 10 (2–135) hours (*p* = 0.555, Mann–Whitney U Test). A disturbance of the pupillary function on admission occurred in 21 (51.2%) younger patients (≤65 years) and in 2 (13.3%) older patients (>65 years) (*p* = 0.014, Fisher Exact Test) (Table 2).

Detected by the paramedics and the emergency doctor at first contact, the mean value for GCS eye opening, motor skills, and verbal response were 2.5 (SD: 1.30) with a median of 3 (1–4), 3.8 (SD: 2.11) with a median of 4 (1–6), and 2.3 (SD: 1.43) with a median of 2 (1–5). The GCS value on admission had a mean 5.7 (SD: 4.16) with a median of 3 (3–15). The mean GCS for younger patients (≤65 years) was 5.1 (SD: 3.76) with a median of 3 (3–15), and for older patients (>65 years), it was 7.4 (SD: 4.89) with a median of 4 (3–15) (*p* = 0.099, Mann–Whitney U Test) (Table 2).

The dominant/left hemisphere was affected in 31 (55.4%) patients (29 right-handed, 2 left-handed patients). In the group of younger patients (≤65 years), bleeding occurred in the dominant hemisphere 24 (58.5%) times and in the group of older patients (>65 years) 7 (46.7%) times (*p* = 0.547). A haematoma evacuation in addition to the DC was performed in 43 (76.8%) of the cases. In the group of younger patients (≤65 years), the additional haematoma evacuation took place in 30 (73.2%) and in the group of older patients (>65 years) in 13 (86.7%) patients (*p* = 0.477, Fisher Exact Test) (Table 2).

There were 22 (39.3%) lobar and 34 (60.7) deep haemorrhages. In the younger group (≤65 years), there were 9 (22%) patients with lobar and 32 (78%) patients with deep haemorrhages. In the older group (>65 years), there were 13 (86.7%) patients with lobar and 2 (13.3%) patients with deeper haemorrhages (*p* < 0.001, Fisher Exact Test). The detailed analysis is depicted in Table 2.

**Figure 1:**
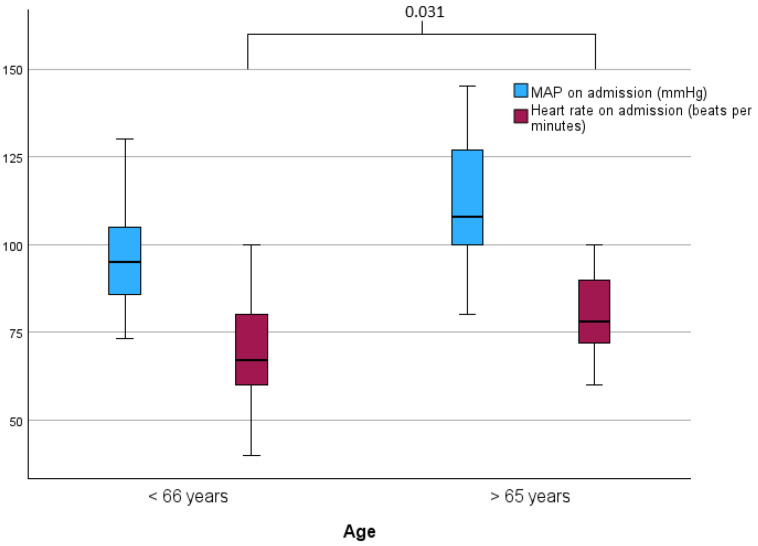
Differences in mean arterial pressure (MAP) and heart rate on admission between the age groups.

**Figure 2:**
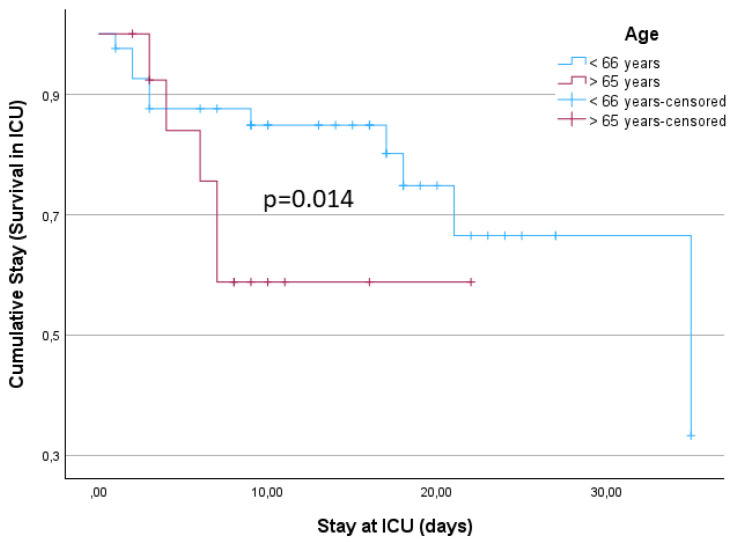
Length of stay at the intensive care unit (ICU) in days.

The mean arterial pressure (MAP) at the emergency room was 103.2 (SD: 18.85) and the mean heart rate was 71.6 (SD: 14.21). The subgroup analysis is depicted in Table 2. The mean heart rate for the younger patients (≤65 years) was significantly lower than in the older group (*p* = 0.031, Mann–Whitney U Test, Figure 1, Table 2). The length of stay in the intensive care unit was a mean 13.0 (SD: 8.43) days. Younger patients (≤65 years) stayed for a mean 14.6 (SD: 8.71) days and older patients (>65 years) stayed for a mean 8.3 (SD: 5.41) days at the intensive care unit (*p* = 0.014, Mann–Whitney U Test) (Figure 2, Table 2).

The outcome according to the GOS is shown in Table 3. Approximately two-thirds (60.7%) showed a moderate (GOS IV and III) and 19.6% a fatal outcome (GOS I) at discharge. The proportion of those who died rose to 30.9% after 12 months. Approximately 6% and 18% had a very good and a good outcome after 12 months, respectively. Approximately 36% had a GOS Grade III and were therefore dependent on support in daily activities. After 12 months, there was no significant difference between younger patients (≤65 years) and older patients (>65 years) after ICH and DC (*p* = 0.328, Kruskal–Wallis Test). Nevertheless, in the group of younger patients (≤65 years), 7.5% had a very good and 15% had a good outcome. There was no good recovery in the group of older patients (>65 years). In summary, after 12 months, 76.4% achieved an unfavourable and 23.7% a favourable outcome. In the group of younger patients (≤65 years), the proportion of patients with a favourable outcome was 22.5%. In the group of older patients (>65 years), however, only 26.7% had a favourable outcome (*p* = 0.501, Fisher Exact Test) (Supplement Figure S1, Table 3).

The regression analysis with the variables gender, age group, time between the onset of symptoms and the intervention, the occurrence of pupillary dysfunction, the GCS on admission, the additional haematoma evacuation, location of bleeding (including lobar and deep position), the bleeding on the dominant side, the MAP and RR on admission, and the ventilation hours resulted in, in univariate and multivariate approach, no significant predictor. The additionally calculated factor of a performed cranioplasty also showed no significant influence on the outcome.

## 4. Discussion

We investigated the outcome after ICH and DC in older adults. Twelve months after ICH and DC, approximately 31% of the patients had died and approximately half of the patients (46%) were depending on support in everyday life. Approximately 24% had a moderate disability or good recovery (GOS > III). In the group of older adults (>65 years), approximately 27% showed a favourable outcome (GOS IV). The risk of an unfavourable outcome after ICH treated by microsurgical evacuation of the haematoma and DC is very high in patients >65 years of age (73%). In our cohort, the time between the onset of symptoms and surgery, the GCS on admission, and the impact on the dominant hemisphere, as well as an additional evacuation of the bleeding, had no impact on outcome.

### 4.1. Stroke, Demographic Change, and Intracerebral Haemorrhage

Stroke is the second leading cause of premature death [1]. About half of all those who die from stroke suffer a haemorrhagic stroke [25,26]. The rate of deaths from stroke after 30 days is given as 25% to 51% [27,28,29]. Further, it is expected that life expectancy will continue to rise in many (high-income) countries and that people will get older and live longer [30]. In Europe, the proportion of people with an age of >60 years is rising steadily [31,32]. For diseases with a strong relationship to age, this means a change in the incidence, with a steady increase [31]. The incidence of ICH increases with age [9,10,11]. It is therefore to be expected that the incidence of ICH as an age-related disease will increase. There are also age-related differences in the characteristics and location of ICH. It is known that younger patients often have deep-seated ICH (cerebellum, basal ganglia, thalamus, pons, internal capsule, etc.) and older patients more often have lobar ICH (cortical or subcortical) [33,34,35,36]. This distribution also applies to our study and is due to the different pathophysiology with lipohyalinosis of the small cerebral vessels, submillimetre Bouchard microaneurysms, and hypertensive genesis in younger patients and the development of peripheral amyloid angiopathy and subsequent vascular fragility in older patients [33]. In the literature, a higher mortality rate is attributed to deep than to lobar ICH [35,37]. However, this last statement is still controversial. Among other studies, the Fast Trail showed that lobar ICH is usually associated with a larger haematoma volume, is more likely to lead to neurological deterioration, and has a worse outcome than deep ICH [38,39]. Even within the group of patients with lobar ICH, Kuramatsu et al. were able to show that there is a relationship between age and haematoma volume. On average, ICH volume is greater in patients aged >70 years than in younger patients [36].

### 4.2. Treatment of Intracerebral Haemorrhage

In addition to medical (non-surgical) and drug therapy approaches, surgical approaches to reduce the intracerebral haematoma have always played a clinical role [21]. With the introduction and spread of computed tomography, ICH could be better localised and determined in size [40,41]. These are possible influences on the fact that the outcome after surgical treatment of ICH has improved. DC, for example, plays a role in a variety of ICH treatment options [21,42]. There are numerous studies of ICH that describe the use of a craniotomy [43,44] or DC [43,44,45,46,47,48,49,50,51,52,53,54,55,56,57,58,59,60] in addition to haematoma evacuation. DC never really showed a clear superiority over the craniotomy for functional outcome (Supplement Figure S1, Table 4). Nevertheless, STICH I and STICH II studies suggested that the surgical approach with craniotomy can show a small but clinically relevant benefit over the non-surgical approach [61,62]. Current surgical approaches tend to pursue minimally invasive procedures to reduce the haematoma size, e.g., by placing a catheter in the middle of the haematoma and administering 1.0 mg tPA every 8 h and up to 9 doses within 3 days (MISTIE III–Minimally Invasive Surgery Plus RT-PA for ICH Evacuation, Phase III) [63], application of plasminogen activator inside the clot [64], usage of sonothrombolysis [65], haematoma-aspiration via obturator over several hours (ENRICH–Early MiNimally-invasive Removal of IntraCerebral Haemorrhage) [66,67,68], or endoscopic aspiration with a special device for evacuation of the ICH (MIND–Artemis in the Removal of Intracerebral haemorrhage; DIST—Dutch Intracerebral Haemorrhage Surgery Trail) [69,70]. However, until now there have not been any surgical approaches that were convincing for a better outcome [44,71].

### 4.3. The Older Adult and Surgical Therapy of Intracerebral Haemorrhage

The volume of the haematoma and above all the space-occupying effect of the haematoma have an impact on the ICP and cerebral perfusion and thus on the outcome after ICH [72,73,74]. DC is able to decrease refractory ICP and additively is able to reduce the space-occupying intracranial effects such as brainstem compression, basal cistern compression, and midline shift [17,75,76]. For this reason, its use in stroke and intracranial haemorrhages with and without haematoma evacuation has been described (Table 4) [43,44,45,46,47,48,49,50,51,52,53,54,55,56,57,58,59,60,74,77,78,79]. In contrast to ischemic stroke, hardly any data are available for ICH and the use of DC in older adults [80]. Murthy et al. included 12 patients, of whom 11 (92%) were discharged surviving. Nine (75%) were ≤65 years old. Three (25%) patients were ≥65 years [70,76]. After the mean follow-up period of 17 (range: 2–39) months, the older patients achieved modified Rankin scores of 2, 4, and 6 [57,81]. Hayes et al. carried out a retrospective study with a total of 51 patients in whom they performed microsurgical haematoma evacuation and DC [43]. There was no significant difference in 30-day mortality between the DC and craniotomy group. This study considered DC mostly for the younger patients. The authors described that they had discovered a strong trend for lowering the probability of poor outcomes (mRS > 3) for patients after DC [43]. Detailed findings on the use of DC in older patients after ICH, however, cannot be drawn from this study. Takeuchi et al. conducted a retrospective study of 21 patients with an average age of 56.6 years (range: 22 to 75 years), on whom they performed an evacuation of the haematoma and DC [45]. There was no significant difference in age, but there was one in the GCS shortly before surgery (unfavourable outcome GCS = 6.1, SD: 2.8 and favourable outcome GCS = 8.8 SD: 2.5; *p* = 0.029). The unfavourable outcome predominates mostly in older patients. Moussa and Khedr examined the outcome after evacuation of the haematoma and DC in one group, and after haematoma evacuation and craniotomy in the other group [46]. After 3 and 6 months, the favourable outcome (GOS = V) occurred in 70% of the DC group and 20% in the craniotomy group. Moderate disability (GOS = IV) occurred in 20% of both groups, and unfavourable (poor) outcome (GOS = I–III) occurred in 10% of the DC group and 60% in the craniotomy group. At the age of ≥70 years and in the DC group, patients achieved moderate and poor outcomes. In the craniotomy group, all elderly patients had poor outcomes. However, no significant difference in outcome could be found for the elderly. Kim et al. examined the outcome in 139 (53%) patients after craniotomy and 125 (47%) patients after DC additively to the evacuation of the haematoma [53]. They found no significant differences in terms of 30-day mortality. After 12 months, 44 (32%) patients in the craniotomy group had died and 31 (22%) were severely impaired. In the DC group, 44 (35%) patients died and 41 (33%) were severely impaired. Although Kim et al. gave indications for the 30-day mortality and the 12-month outcome for older patients (>65 years), this is not specified for DC. In the group of older patients (≥65 years), the 30-day mortality was 20%, compared to 9% in the group of younger patients (<65 years). An unfavourable outcome (GOS = I–II) occurred in 69% of older patients (≥65 years) and 53% of younger patients (<65 years) [53]. Whether a DC influences survival and functional outcome cannot be determined from this study (Table 4).

Studies have also been published in which the surgical treatment of the ICH consisted only of a DC without evacuation of the haematoma [48,52]. However, no detailed information can be obtained for older adults from these studies (Table 3).

The question of whether cranioplasty after ICH and DC improves the functional outcome depending on age has not yet been addressed in a large number of patients and prospective approach. This hypothesis is evaluated positively after TBI and DC, but shows no reliable data after ICH and DC [82,83].

In summary, the presented literature shows that the outcome after DC in ICH is predominantly unfavourable. DC takes longer than a standard craniotomy [44]. However, survival becomes more likely with DC [56,57]. There is little knowledge about the application of DC at ICH in an aging social group. Nevertheless, the results so far show that if DC is necessary in the therapy of ICH, older adults have a very high risk of unfavourable outcome. Despite progress, this risk does not seem to have changed much over the decades. Nevertheless, most of the recent studies are nonrandomised and represent small sample sizes with a large proportion of patients with poor outcomes [52]. This fact also applies to our retrospective multicentre study. This leads to an underpowered status, which makes it difficult to detect differences in clinically meaningful measures such as mortality and neurological morbidity. Most cases of older patients represent a selected patient population [57]. Secondary diagnoses such as cancer, severe metabolic diseases, kidney or liver diseases, or dementia are rarely included in long-term considerations [46]. Due to the limited amount of prospective data, the results of the SWITCH study (Decompressive Hemicraniectomy in Intracerebral Haemorrhage) remain to be seen [18]. Prospective randomised studies must prove whether new, less invasive surgical approaches lead to an improvement in the functional outcome in ICH, especially for older adults.

### 4.4. Limitations

Its retrospective character as well as the small number of patients are the main limitations of our study. Furthermore, the indication for decompressive craniectomy in the case of ICH possesses a potential bias, since the indication criteria are in some cases difficult to define clearly and depend on experience and surgeons’ preferences.

## 5. Conclusions

In the majority of cases, patients >65 years of age who are treated with microsurgical haematoma evacuation and DC after ICH have a poor outcome. This represents a similar result to the younger patients. However, in the long term, only a few older adults (>65 years of age) had a favourable outcome after this procedure. Nevertheless, older patients may achieve a favourable clinical outcome after DC and ICH. At this moment, we lack precise identification of early predictors.

## Figures and Tables

**Table 1:** Patients’ demographic data.

	Total	*p*	≤65 Years of Age	>65 Years of Age	*p*
*N* (%)	56 (100)		41 (73.2)	15 (26.8)	<0.001
Women (%)	23 (41.1)	<0.001	15 (36.6)	8 (53.3)	0.359
Men (%)	33 (58.9)		26 (63.4)	7 (46.7)	
Mean age (SD)	53.3 (16.13)		46.8 (13.19)	71.3 (7.28)	<0.001
Median (Range)	56 (7–89)		50 (7–64)	69 (66–89)	
**Age**					
Mean Women (SD)	54.6 (16.50)	0.247	47.3 (16.21)	68.3 (2.05)	<0.001
Median Women (Range)	59 (7–71)		52 (7–64)	68 (66–71)	
Mean Men (SD)	52.4 (16.05)		46.4 (11.45)	74.7 (9.62)	<0.001
Median Men (Range)	50 (23–89)		49 (23–63)	69 (66–89)	

**Table 2:** Patients’ clinical and radiological characteristics.

	Total (%)	≤65 Years of Age	>65 Years of Age	*p*
*N* (%)	56 (100)	41 (73.2)	15 (26.8)	<0.001
In-Hospital death	15 (26.8)	10 (24.4)	5 (33.3)	0.514
Time between symptoms and surgery (hours)				
Mean (SD)	39.6 (63.66)	44.4 (70.79)	27.9 (41.70)	0.555
Median (Range)	13 (2–288)	14 (3–288)	10 (2–135)	
Pupillary dysfunction				
*N* (%)	23 (41.1)	21 (51.2)	2 (13.3)	0.014
Glasgow Coma Score on admission				
Mean (SD)	5.7 (4.16)	5.1 (3.76)	7.4 (4.89)	0.099
Median (Range)	3 (3–15)	3 (3–15)	4 (3–15)	
Eye opening				
Mean (SD)	1.6 (1.11)	1.5 (1.06)	2.0 (1.23)	0.102
Median (Range)	1 (1–4)	1 (1–4)	1 (1–4)	
Motor Skills				
Mean (SD)	2.4 (1.99)	2.1 (1.76)	3.2 (2.39)	0.072
Median (Range)	1 (1–6)	1 (1–6)	2 (1–6)	
Verbal response				
Mean (SD)	1.6 (1.27)	1.4 (1.11)	2.2 (1.57)	0.043
Median (Range)	1 (1–5)	1 (1–5)	1 (1–5)	
Dominant hemisphere				
affected	31 (55.4)	24 (58.5)	7 (46.7)	0.547
Evacuation of the haematoma				
yes	43 (76.8)	30 (73.2)	13 (86.7)	0.477
no	13 (23.2)	11 (26.8)	2 (13.3)	
MAP (mmHg) on admission				
Mean (SD)	103.2 (18.85)	100.0 (17.00)	112.9 (21.80)	0.104
Heart rate (bpm) on admission				
Mean (SD)	71.6 (14.21)	68.6 (14.01)	80.0 (11.58)	0.031
Length of stay at ICU(days)				
Mean (SD)	13.0 (8.43)	14.6 (8.71)	8.3 (5.41)	0.014
Haematoma characteristics				
Volume in cm^3^				
Mean (SD)	68.7 (33.75)	68.6 (33.16)	69.1 (36.51)	0.890
Median (Range)	58.4 (19.3–161.9)	58.8 (19.3–148.0)	56.4 (27.6–161.9)	
Midline shift in mm				
Mean (SD)	8.0 (4.16)	7.7 (4.20)	8.7 (4.11)	0.313
Median (Range)	7.1 (2.0–20.0)	6.1 (2.0–20.0)	8.1 (2.0–16.1)	
Occurrence of intraventricular haemorrhage, *N* (%)				
Yes	30 (53.6)	23 (56.1)	7 (46.7)	0.560
Lobar and deep localization				
Lobar, *N* (%)	22 (39.3)	9 (22)	13 (86.7)	<0.001
Deep, *N* (%)	34 (60.7)	32 (78)	2 (13.3)	

**Table 3:** Outcome after discharge and after 12 months.

	*N* (%)	≤65 Years of Age	>65 Years of Age	*p*
	56 (100)	41 (73.2)	15 (26.8)	<0.001
Glasgow Outcome Scale at discharge				
Grade I	11 (19.6)	7 (17.1)	4 (26.7)	0.911
Grade II	11 (19.6)	10 (24.4)	1 (6.7)	
Grade III	30 (53.6)	21 (51.2)	9 (60)	
Grade IV	4 (7.1)	3 (7.3)	1 (6.7)	
Grade V	0 (0)	0 (0)	0 (0)	
Glasgow Outcome Scale after 12 months (one patient lost in follow-up)				
Grade I	17 (30.9)	10 (25)	7 (46.7)	0.328
Grade II	5 (9.1)	4 (10)	1 (6.7)	
Grade III	20 (36.4)	17 (42.5)	3 (20)	
Grade IV	10 (18.2)	6 (15)	4 (26.7)	
Grade V	3 (5.5)	3 (7.5)	0 (0)	
Outcome after 12 months (one patient lost in follow-up)				
Unfavourable Outcome	42 (76.4)	31 (77.5)	11 (73.4)	0.501
Favourable Outcome	13 (23.7)	9 (22.5)	4 (26.7)	

**Table 4:** Findings of the reviewed literature (NA = not applicable).

Authors	Country	Research Design	Total No. of Patients	No. of Patients with DC	Figures for Age (Years)	Proportion of Patients (>65 Years)	DC Alone/DC with Haematoma Evacuation	Follow-Up Period (Months)	Mortality Rate	Proportion of Unfavourable Outcome	Proportion of Older Adults (>65 Years) with Unfavourable Outcome	Significant Results for the Outcome
Dierssen et al. (1983) [49]	Spain	retrospective	73	73	Ø52	6 (8.2%)	0%/100%	24	33%	38%	4 (67%)	Mortality was influenced by clinical course, time interval between onset of symptoms and surgery, brainstem dysfunction, and age
Esquenazi et al. (2015) [48]	USA	retrospective, prospective database	73	73	Ø52	NA	14%/86%	3	27%	71%	NA	GOS was influenced by GCS at admission, affected left hemisphere, and ICH volume
Fung et al. (2012) [50]	Switzerland	retrospective, matched case-control	27	12	~x 48/51	NA	100%/0%	6	25% for DC group	50% for DC group	NA	
Gildersleeve et al. (2019) [51]	USA	retrospective, prospective database, matched case-control	86	43	Ø49/52	NA	23%/77%	3	21%	74%	NA	
Hayes et al. (2013) [43]	USA	retrospective,	51	18	Ø48/57.5	NA	0%/100%	1	29%	64%	NA	
Hedge et al. (2020) [44]	India	retrospective, case-control	132	54	Ø51/53	NA	0%/100%	3	24%	80%	NA	Patients with lobar haematomas had better outcomes than those with deep-seated haematomas
Heuts et al. (2013) [52]	USA	prospective, group comparison	154	5	~x 68.5/47/43	0	3%/1%	6	20% for DC only	60% for DC only	NA	
Kim et al. (2018) [53]	Korea	retrospective, group comparison prospective database	264	125	Ø63/64	NA	0%/100%	12	35% for DC group	68% for DC group	NA	Prognostic preoperative factors: age (<65 years), no intraventricular haemorrhage, and GCS ≥ 9, male sex, right hemisphere, haematoma volume, less midline shift, ICH score, short time to surgery
Ling et al. (2021) [55]	China	retrospective, group comparison	290	152	Ø59/60	NA	0%/52%	6	3% for DC group	24% for DC group	NA	
Lo et al. (2017) [56]	Singapore	Retrospective, matched case-control	126	54	~x 55/58	NA	9%/91%	12	38% for DC group	87%	NA	Better survival rate with DC but no difference in functional outcome compared with best medical treatment
Moussa and Khedr (2017) [46]	Egypt	prospective, randomised group comparison	40	20	Ø59	18% >69 years	0%/50%	6	10% for DC group	30%	4 patients in DC group with ≥70 years 10% moderate, 10% poor	Better outcome in younger patients (≤70 years), higher admission GCS, subcortical location and smaller size of haematoma, less midline shift
Murthy et al. (2005) [57]	India	retrospective	12	12	Ø50	25%	0%/100%	Ø 17	8%	42%	17%	
Ramnarayan et al. (2009) [58]	India	retrospective	23	23	31 to 68	NA	100%/0%	3	13%	43% (GOS I–IV)	NA	
Rasras et al. (2018) [59]	Iran	Prospective randomised, group comparison	30	13	Ø60/59	NA	43%/0%	6	30%; 13% for DC group	67%; 69% in DC group		DC without clot evacuation may produce the same results like craniotomy and clot evacuation
Satter et al. (2016) [60]	Bangladesh	prospective, group comparison	80	40	Ø59/58	NA	100%/0%	3	38%; 33% for DC group	81%; 75% for DC group		

## Data Availability

Data can be provided by the corresponding author on request.

## Supplementary Materials

The following supporting information can be downloaded at: https://www.mdpi.com/article/10.3390/neurolint16030044/s1, Supplement Figure S1: Survival and functional outcome at discharge and after 12 months according to the Glasgow Outcome Scale (GOS); Favourable Outcome (GOS IV and V), Unfavourable Outcome (GOS I to III).

## References

[1] GBD 2016 Stroke Collaborators (2019). Global, regional, and national burden of stroke, 1990–2016: A systematic analysis for the Global Burden of Disease Study 2016. Lancet Neurol..

[2] Rincon F., Mayer S.A. (2013). The epidemiology of intracerebral hemorrhage in the United States from 1979 to 2008. Neurocrit. Care.

[3] Ikram M.A., Wieberdink R.G., Koudstaal P.J. (2012). International epidemiology of intracerebral hemorrhage. Curr. Atheroscler. Rep..

[4] Pedersen T.G.B., Vinter N., Schmidt M., Frost L., Cordsen P., Andersen G., Johnsen S.P. (2022). Trends in the incidence and mortality of intracerebral hemorrhage, and the associated risk factors, in Denmark from 2004 to 2017. Eur. J. Neurol..

[5] Goldstein L.B. (2020). Introduction for Focused Updates in Cerebrovascular Disease. Stroke.

[6] Burke J.F., Skolarus L.E. (2017). Are More Young People Having Strokes?—A Simple Question with an Uncertain Answer. JAMA Neurol..

[7] Hathidara M.Y., Saini V., Malik A.M. (2019). Stroke in the Young: A Global Update. Curr. Neurol. Neurosci. Rep..

[8] Feigin V.L., Lawes C.M., Bennett D.A., Barker-Collo S.L., Parag V. (2009). Worldwide stroke incidence and early case fatality reported in 56 population-based studies: A systematic review. Lancet Neurol..

[9] Jolink W.M., Klijn C.J., Brouwers P.J., Kappelle L.J., Vaartjes I. (2015). Time trends in incidence, case fatality, and mortality of intracerebral hemorrhage. Neurology.

[10] Stein M., Misselwitz B., Hamann G.F., Scharbrodt W., Schummer D.I., Oertel M.F. (2012). Intracerebral hemorrhage in the very old: Future demographic trends of an aging population. Stroke.

[11] Khellaf M., Quantin C., d’Athis P., Fassa M., Jooste V., Hervieu M., Giroud M., Bejot Y. (2010). Age-period-cohort analysis of stroke incidence in Dijon from 1985 to 2005. Stroke.

[12] An S.J., Kim T.J., Yoon B.W. (2017). Epidemiology, Risk Factors, and Clinical Features of Intracerebral Hemorrhage: An Update. J. Stroke.

[13] Pol L.G., Tymkiw D.R. (1990). Demographic change and the supply of physicians, hospitals, and hospital beds: Marketing implications. J. Hosp. Mark..

[14] Seferian E.G., Afessa B. (2006). Demographic and clinical variation of adult intensive care unit utilization from a geographically defined population. Crit. Care Med..

[15] Smith S., Horgan F., Sexton E., Cowman S., Hickey A., Kelly P., McGee H., Murphy S., O’Neill D., Royston M. (2013). The future cost of stroke in Ireland: An analysis of the potential impact of demographic change and implementation of evidence-based therapies. Age Ageing.

[16] Jennett B., Bond M. (1975). Assessment of outcome after severe brain damage. Lancet.

[17] Kapapa T., Brand C., Wirtz C.R., Woischneck D. (2016). Outcome after Decompressive Craniectomy in Different Pathologies. World Neurosurg..

[18] Greenberg S.M., Ziai W.C., Cordonnier C., Dowlatshahi D., Francis B., Goldstein J.N., Hemphill J.C., Johnson R., Keigher K.M., Mack W.J. (2022). 2022 Guideline for the Management of Patients with Spontaneous Intracerebral Hemorrhage: A Guideline from the American Heart Association/American Stroke Association. Stroke.

[19] Patel S., Maria-Rios J., Parikh A., Okorie O.N. (2023). Diagnosis and management of elevated intracranial pressure in the emergency department. Int. J. Emerg. Med..

[20] Harder T.J., Leary O.P., Yang Z., Lucke-Wold B., Liu D.D., Still M.E.H., Zhang M., Yeatts S.D., Allen J.W., Wright D.W. (2023). Early Signs of Elevated Intracranial Pressure on Computed Tomography Correlate with Measured Intracranial Pressure in the Intensive Care Unit and Six-Month Outcome after Moderate to Severe Traumatic Brain Injury. J. Neurotrauma.

[21] de Oliveira Manoel A.L. (2020). Surgery for spontaneous intracerebral hemorrhage. Crit. Care.

[22] Al-Jishi A., Saluja R.S., Al-Jehani H., Lamoureux J., Maleki M., Marcoux J. (2011). Primary or secondary decompressive craniectomy: Different indication and outcome. Can. J. Neurol. Sci..

[23] Robba C., Iannuzzi F., Taccone F.S. (2021). Tier-three therapies for refractory intracranial hypertension in adult head trauma. Minerva. Anestesiol..

[24] Aguilar M.I., Brott T.G. (2011). Update in intracerebral hemorrhage. Neurohospitalist.

[25] van Asch C.J., Luitse M.J., Rinkel G.J., van der Tweel I., Algra A., Klijn C.J. (2010). Incidence, case fatality, and functional outcome of intracerebral haemorrhage over time, according to age, sex, and ethnic origin: A systematic review and meta-analysis. Lancet Neurol..

[26] Carlsson M., Wilsgaard T., Johnsen S.H., Johnsen L.H., Lochen M.L., Njolstad I., Mathiesen E.B. (2021). Long-Term Survival, Causes of Death, and Trends in 5-Year Mortality after Intracerebral Hemorrhage: The Tromso Study. Stroke.

[27] Safatli D.A., Gunther A., Schlattmann P., Schwarz F., Kalff R., Ewald C. (2016). Predictors of 30-day mortality in patients with spontaneous primary intracerebral hemorrhage. Surg. Neurol. Int..

[28] Fogelholm R., Murros K., Rissanen A., Avikainen S. (2005). Long term survival after primary intracerebral haemorrhage: A retrospective population based study. J. Neurol. Neurosurg. Psychiatry.

[29] Malinova V., Iliev B., Mielke D., Rohde V. (2019). Intracerebral Hemorrhage-Score Allows a Reliable Prediction of Mortality in Patients with Spontaneous Intracerebral Hemorrhage Managed by Fibrinolytic Therapy. Cerebrovasc. Dis..

[30] Kontis V., Bennett J.E., Mathers C.D., Li G., Foreman K., Ezzati M. (2017). Future life expectancy in 35 industrialised countries: Projections with a Bayesian model ensemble. Lancet.

[31] Giampaoli S. (2000). Epidemiology of major age-related diseases in women compared to men. Aging.

[32] Eurostat Population Structure and Ageing. https://ec.europa.eu/eurostat/statistics-explained/index.php?title=Population_structure_and_ageing.

[33] O’Carroll C.B., Brown B.L., Freeman W.D. (2021). Intracerebral Hemorrhage: A Common yet Disproportionately Deadly Stroke Subtype. Mayo Clin. Proc..

[34] Labovitz D.L., Halim A., Boden-Albala B., Hauser W.A., Sacco R.L. (2005). The incidence of deep and lobar intracerebral hemorrhage in whites, blacks, and Hispanics. Neurology.

[35] Lioutas V.A., Beiser A.S., Aparicio H.J., Himali J.J., Selim M.H., Romero J.R., Seshadri S. (2020). Assessment of Incidence and Risk Factors of Intracerebral Hemorrhage among Participants in the Framingham Heart Study between 1948 and 2016. JAMA Neurol..

[36] Kuramatsu J.B., Sauer R., Mauer C., Lucking H., Kloska S.P., Kiphuth I.C., Staykov D., Kohrmann M., Huttner H.B. (2011). Correlation of age and haematoma volume in patients with spontaneous lobar intracerebral haemorrhage. J. Neurol. Neurosurg. Psychiatry.

[37] Samarasekera N., Fonville A., Lerpiniere C., Farrall A.J., Wardlaw J.M., White P.M., Smith C., Al-Shahi Salman R., Lothian Audit of the Treatment of Cerebral Haemorrhage Collaborators (2015). Influence of intracerebral hemorrhage location on incidence, characteristics, and outcome: Population-based study. Stroke.

[38] Kuohn L.R., Witsch J., Steiner T., Sheth K.N., Kamel H., Navi B.B., Merkler A.E., Murthy S.B., Mayer S.A. (2022). Early Deterioration, Hematoma Expansion, and Outcomes in Deep versus Lobar Intracerebral Hemorrhage: The FAST Trial. Stroke.

[39] Falcone G.J., Biffi A., Brouwers H.B., Anderson C.D., Battey T.W., Ayres A.M., Vashkevich A., Schwab K., Rost N.S., Goldstein J.N. (2013). Predictors of hematoma volume in deep and lobar supratentorial intracerebral hemorrhage. JAMA Neurol..

[40] Pineda A. (1977). Computed tomography in intracerebral hemorrhage. Surg. Neurol..

[41] Li N., Wang Y., Wang W., Ma L., Xue J., Weissenborn K., Dengler R., Worthmann H., Wang D.Z., Gao P. (2011). Contrast extravasation on computed tomography angiography predicts clinical outcome in primary intracerebral hemorrhage: A prospective study of 139 cases. Stroke.

[42] Kirshner H., Schrag M. (2021). Management of Intracerebral Hemorrhage: Update and Future Therapies. Curr. Neurol. Neurosci. Rep..

[43] Hayes S.B., Benveniste R.J., Morcos J.J., Aziz-Sultan M.A., Elhammady M.S. (2013). Retrospective comparison of craniotomy and decompressive craniectomy for surgical evacuation of nontraumatic, supratentorial intracerebral hemorrhage. Neurosurg. Focus.

[44] Hegde A., Prasad G.L., Menon G. (2020). Decompressive Craniectomy in Spontaneous Intracerebral Hemorrhage: A Comparison with Standard Craniotomy Using Propensity-Matched Analysis. World Neurosurg..

[45] Takeuchi S., Wada K., Nagatani K., Otani N., Mori K. (2013). Decompressive hemicraniectomy for spontaneous intracerebral hemorrhage. Neurosurg. Focus.

[46] Moussa W.M., Khedr W. (2017). Decompressive craniectomy and expansive duraplasty with evacuation of hypertensive intracerebral hematoma, a randomized controlled trial. Neurosurg. Rev..

[47] Yao Z., Ma L., You C., He M. (2018). Decompressive Craniectomy for Spontaneous Intracerebral Hemorrhage: A Systematic Review and Meta-analysis. World Neurosurg..

[48] Esquenazi Y., Savitz S.I., El Khoury R., McIntosh M.A., Grotta J.C., Tandon N. (2015). Decompressive hemicraniectomy with or without clot evacuation for large spontaneous supratentorial intracerebral hemorrhages. Clin. Neurol. Neurosurg..

[49] Dierssen G., Carda R., Coca J.M. (1983). The influence of large decompressive craniectomy on the outcome of surgical treatment in spontaneous intracerebral haematomas. Acta Neurochir..

[50] Fung C., Murek M., Z’Graggen W.J., Krahenbuhl A.K., Gautschi O.P., Schucht P., Gralla J., Schaller K., Arnold M., Fischer U. (2012). Decompressive hemicraniectomy in patients with supratentorial intracerebral hemorrhage. Stroke.

[51] Gildersleeve K.L., Hirzallah M.I., Esquenazi Y., Moomaw C.J., Sekar P., Cai C., Tandon N., Woo D., Gonzales N.R. (2019). Hemicraniectomy for Supratentorial Primary Intracerebral Hemorrhage: A Retrospective, Propensity Score Matched Study. J. Stroke Cerebrovasc. Dis..

[52] Heuts S.G., Bruce S.S., Zacharia B.E., Hickman Z.L., Kellner C.P., Sussman E.S., McDowell M.M., Bruce R.A., Connolly E.S. (2013). Decompressive hemicraniectomy without clot evacuation in dominant-sided intracerebral hemorrhage with ICP crisis. Neurosurg. Focus.

[53] Kim D.B., Park S.K., Moon B.H., Cho B.R., Jang D.K., Jang K.S. (2018). Comparison of craniotomy and decompressive craniectomy in large supratentorial intracerebral hemorrhage. J. Clin. Neurosci..

[54] Lele A.V., Fong C.T., Newman S.F., O’Reilly-Shah V., Walters A.M., Athiraman U., Souter M.J., Levitt M.R., Vavilala M.S. (2023). Anesthesiology Performance Improvement and Reporting Exchange (ASPIRE) Quality Metrics in Patients Undergoing Decompressive Craniectomy and Endoscopic Clot Evacuation after Spontaneous Supratentorial Intracerebral Hemorrhage: A Retrospective Observational Study. J. Neurosurg. Anesth..

[55] Ling M., Zhang Q., Zang L., Li X., Liu Q. (2021). Decompressive craniectomy can improve the recovery of neurological function, daily living ability and life quality of patients with intracerebral hemorrhage after surgery. Am. J. Transl. Res..

[56] Lo Y.T., See A.A.Q., King N.K.K. (2017). Decompressive Craniectomy in Spontaneous Intracerebral Hemorrhage: A Case-Control Study. World Neurosurg..

[57] Murthy J.M., Chowdary G.V., Murthy T.V., Bhasha P.S., Naryanan T.J. (2005). Decompressive craniectomy with clot evacuation in large hemispheric hypertensive intracerebral hemorrhage. Neurocrit. Care.

[58] Ramnarayan R., Anto D., Anilkumar T.V., Nayar R. (2009). Decompressive Hemicraniectomy in Large Putaminal Hematomas: An Indian Experience. J. Stroke Cerebrovasc. Dis..

[59] Rasras S., Safari H., Zeinali M., Jahangiri M. (2018). Decompressive hemicraniectomy without clot evacuation in supratentorial deep-seated intracerebral hemorrhage. Clin. Neurol. Neurosurg..

[60] Satter A.R., Islam M.R., Haque M.R., Mahmood E., Rahman M.Z., Barman N., Rahman M.A. (2016). Comparison between Decompressive Craniectomy with Durotomy and Conservative Treatment in Spontaneous Supratentorial Intracerebral Hemorrhage. Mymensingh. Med. J..

[61] Mendelow A.D., Gregson B.A., Fernandes H.M., Murray G.D., Teasdale G.M., Hope D.T., Karimi A., Shaw M.D., Barer D.H. (2005). Early surgery versus initial conservative treatment in patients with spontaneous supratentorial intracerebral haematomas in the International Surgical Trial in Intracerebral Haemorrhage (STICH): A randomised trial. Lancet.

[62] Mendelow A.D., Gregson B.A., Rowan E.N., Murray G.D., Gholkar A., Mitchell P.M., Investigators S.I. (2013). Early surgery versus initial conservative treatment in patients with spontaneous supratentorial lobar intracerebral haematomas (STICH II): A randomised trial. Lancet.

[63] Hanley D.F., Thompson R.E., Rosenblum M., Yenokyan G., Lane K., McBee N., Mayo S.W., Bistran-Hall A.J., Gandhi D., Mould W.A. (2019). Efficacy and safety of minimally invasive surgery with thrombolysis in intracerebral haemorrhage evacuation (MISTIE III): A randomised, controlled, open-label, blinded endpoint phase 3 trial. Lancet.

[64] Naff N., Williams M.A., Keyl P.M., Tuhrim S., Bullock M.R., Mayer S.A., Coplin W., Narayan R., Haines S., Cruz-Flores S. (2011). Low-dose recombinant tissue-type plasminogen activator enhances clot resolution in brain hemorrhage: The intraventricular hemorrhage thrombolysis trial. Stroke.

[65] Newell D.W., Shah M.M., Wilcox R., Hansmann D.R., Melnychuk E., Muschelli J., Hanley D.F. (2011). Minimally invasive evacuation of spontaneous intracerebral hemorrhage using sonothrombolysis. J. Neurosurg..

[66] Labib M.A., Shah M., Kassam A.B., Young R., Zucker L., Maioriello A., Britz G., Agbi C., Day J.D., Gallia G. (2017). The Safety and Feasibility of Image-Guided BrainPath-Mediated Transsulcul Hematoma Evacuation: A Multicenter Study. Neurosurgery.

[67] Bauer A.M., Rasmussen P.A., Bain M.D. (2017). Initial Single-Center Technical Experience With the BrainPath System for Acute Intracerebral Hemorrhage Evacuation. Oper. Neurosurg..

[68] Pradilla G., Ratcliff J.J., Hall A.J., Saville B.R., Allen J.W., Frankel M., Wright D.W., Barrow D.L., ENRICH-Investigators Efficacy and Safety of Early Minimally Invasive Removal of Intracerebral Hemorrhage (ENRICH): A Multicenter Randomized Adaptive Trial. Proceedings of the American Association of Neurological Surgeons Annual Scientific Meeting.

[69] Kobata H., Ikeda N. (2021). Recent Updates in Neurosurgical Interventions for Spontaneous Intracerebral Hemorrhage: Minimally Invasive Surgery to Improve Surgical Performance. Front. Neurol..

[70] Pan J., Chartrain A.G., Scaggiante J., Spiotta A.M., Tang Z., Wang W., Pradilla G., Murayama Y., Mori R., Mocco J. (2020). A Compendium of Modern Minimally Invasive Intracerebral Hemorrhage Evacuation Techniques. Oper. Neurosurg..

[71] Juvela S., Heiskanen O., Poranen A., Valtonen S., Kuurne T., Kaste M., Troupp H. (1989). The treatment of spontaneous intracerebral hemorrhage. A prospective randomized trial of surgical and conservative treatment. J. Neurosurg..

[72] Broderick J.P., Brott T.G., Duldner J.E., Tomsick T., Huster G. (1993). Volume of intracerebral hemorrhage. A powerful and easy-to-use predictor of 30-day mortality. Stroke.

[73] Tuhrim S., Dambrosia J.M., Price T.R., Mohr J.P., Wolf P.A., Hier D.B., Kase C.S. (1991). Intracerebral hemorrhage: External validation and extension of a model for prediction of 30-day survival. Ann. Neurol..

[74] Marinkovic I., Strbian D., Pedrono E., Vekovischeva O.Y., Shekhar S., Durukan A., Korpi E.R., Abo-Ramadan U., Tatlisumak T. (2009). Decompressive craniectomy for intracerebral hemorrhage. Neurosurgery.

[75] Sahuquillo J., Arikan F. (2006). Decompressive craniectomy for the treatment of refractory high intracranial pressure in traumatic brain injury. Cochrane Database Syst. Rev..

[76] Goedemans T., Verbaan D., Coert B.A., Kerklaan B.J., van den Berg R., Coutinho J.M., van Middelaar T., Nederkoorn P.J., Vandertop W.P., van den Munckhof P. (2017). Neurologic Outcome after Decompressive Craniectomy: Predictors of Outcome in Different Pathologic Conditions. World Neurosurg..

[77] Steiner T., Ringleb P., Hacke W. (2001). Treatment options for large hemispheric stroke. Neurology.

[78] Ziai W.C., Port J.D., Cowan J.A., Garonzik I.M., Bhardwaj A., Rigamonti D. (2003). Decompressive craniectomy for intractable cerebral edema: Experience of a single center. J. Neurosurg. Anesth..

[79] Fung C., Murek M., Klinger-Gratz P.P., Fiechter M., Z’Graggen W.J., Gautschi O.P., El-Koussy M., Gralla J., Schaller K., Zbinden M. (2016). Effect of Decompressive Craniectomy on Perihematomal Edema in Patients with Intracerebral Hemorrhage. PLoS ONE.

[80] Robertson F.C., Dasenbrock H.H., Gormley W.B. (2017). Decompressive Hemicraniectomy for Stroke in Older Adults: A Review. J. Neurol. Neuromed..

[81] Wilson J.T., Hareendran A., Hendry A., Potter J., Bone I., Muir K.W. (2005). Reliability of the modified Rankin Scale across multiple raters: Benefits of a structured interview. Stroke.

[82] Mele C., Bassetto A., Boetto V., Nardone A., Pingue V. (2022). Impact of Cranioplasty on Rehabilitation Course of Patients with Traumatic or Hemorrhagic Brain Injury. Brain Sci..

[83] Mee H., Anwar F., Timofeev I., Owens N., Grieve K., Whiting G., Alexander K., Kendrick K., Helmy A., Hutchinson P. (2022). Cranioplasty: A Multidisciplinary Approach. Front. Surg..

