# Supplementary material for: Outcome after Intracerebral Haemorrhage and Decompressive Craniectomy in Older Adults
Source: Neurol Int. 2024 May 20;16(3):590–604. doi: 10.3390/neurolint16030044 (PMC11130851; doi:10.3390/neurolint16030044)
Supplement: Supplementary file 1 [file neurolint-16-00044-s001.zip › neurolint-2955327-supplementary.pdf]

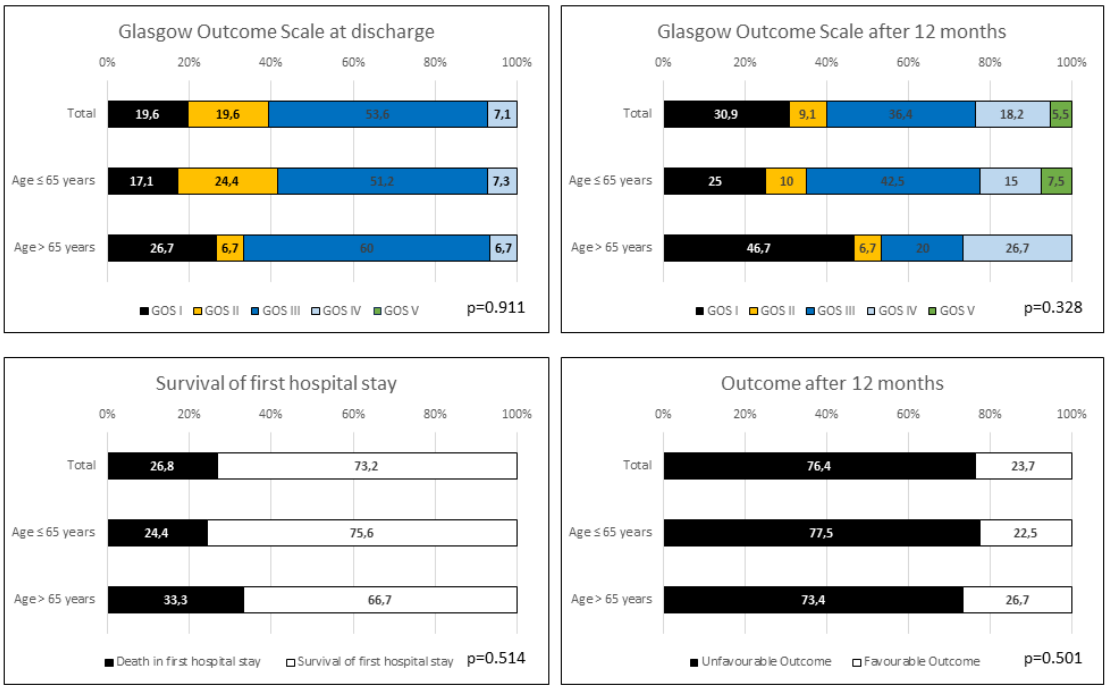

Supplement Figure S1: Survival and functional outcome at discharge and after 12 months according to the Glasgow Outcome Scale (GOS); Favourable Outcome (GOS IV and V), Unfavourable Outcome (GOS I to III).
